# Assessment of Two Crosslinked Polymer Systems Including Hydrolyzed Polyacrylamide and Acrylic Acid–Hydrolyzed Polyacrylamide Co-Polymer for Carbon Dioxide and Formation Water Diversion Through Relative Permeability Reduction in Unconsolidated Sandstone Formation

**DOI:** 10.3390/polym16243503

**Published:** 2024-12-17

**Authors:** Sherif Fakher, Abdelaziz Khlaifat, Karim Mokhtar, Mariam Abdelsamei

**Affiliations:** Department of Petroleum and Energy Engineering, School of Science and Engineering, The American University in Cairo, New Cairo 11835, Egypt; abdelaziz.khlaifat@aucegypt.edu (A.K.); karim.mokhtar2002@aucegypt.edu (K.M.); mariamezz2255@aucegypt.edu (M.A.)

**Keywords:** crosslinked co-polymer, carbon dioxide and formation water diversion, permeability reduction, unconsolidated sandstone formation

## Abstract

One of the most challenging aspects of manipulating the flow of fluids in subsurfaces is to control their flow direction and flow behavior. This can be especially challenging for compressible fluids, such as CO_2_, and for multiphase flow, including both water and carbon dioxide (CO_2_). This research studies the ability of two crosslinked polymers, including hydrolyzed polyacrylamide and acrylic acid/hydrolyzed polyacrylamide crosslinked polymers, to reduce the permeability of both CO_2_ and formation water using different salinities and permeability values and in the presence of crude oil under different injection rates. The result showed that both polymers managed to reduce the permeability of water effectively; however, their CO_2_ permeability-reduction potential was much lower, with the CO_2_ permeability reduction being less than 50% of the water reduction potential in the majority of the experiments. This was mainly due to the high flow rate of the CO_2_ compared to the water, which resulted in significant shearing of the crosslinked polymer. The crosslinked polymers’ swelling ratios were impacted differently based on the salinity, with the maximum swelling ratio being 9.8. The HPAM polymer was negatively affected by the presence of crude oil, whereas increasing salinity improved its performance greatly. All in all, both polymers had a higher permeability reduction for the formation water compared to CO_2_ under all conditions. This research can help improve the applicability of CO_2_-enhanced oil recovery and CO_2_ storage in depleted oil reservoirs. The ability of the crosslinked polymers to improve CO_2_ storage will be a main focus of future research.

## 1. Introduction

One of the most challenging operational problems associated with oil and gas wells is excessive water and non-hydrocarbon gas production. This is even more profound in waterflooding and carbon dioxide (CO_2_) flooding and storage applications [1,2,3,4,5,6,7,8]. Fluid shutoff chemicals are a viable solution to reduce the early breakthrough and excessive production of water and CO_2_ by reducing their permeability in specific zones and diverting their flow to other unflooded zones. One of the most widely used chemicals is crosslinked polymers [9,10,11,12,13,14,15]. Since these polymers have different properties and characteristics, new crosslinked polymers are constantly needed to improve their ability to reduce formation water and CO_2_ permeability effectively.

Different crosslinked chemicals have been used to obtain relative permeability reductions either directly or indirectly. Organic zirconium crosslinkers utilize polynuclear hydroxyl bridge complex ions of zirconium to create gels from linear polymers by forming intermolecular chemical bonds under specific conditions [16,17,18,19,20,21,22]. Recently, there have been significant advancements in the study of crosslinking agents for controlling polymer gel profiles [23,24,25,26]. For instance, Cui J. [27] investigated an organic zirconium gel cross-linking agent, and Bojun [28] employed organic zirconium to cross-link anionic polyacrylamide with a high molecular weight and low concentration to create a water-blocking gel. Assessments of a high-molecular-weight (HMW) organically crosslinked polymer (OCP) gel system, referred to as HMW-OCP, for specific applications have been conducted by Freddy [29]. Their conformance technology arises from crosslinking reactions between HMW polyacrylamide and polyethylenimine (PEI). The moderate viscosities of the fluids, due to the high molecular weight of the system’s components, enable effective gel placement in fractures and high-permeability channels without penetrating the rock matrix. Additionally, a lower-molecular-weight version of this polymer system has been shown to successfully manage water production in matrix applications. Polyacrylamide gels have long been employed to reduce water cuts in production wells and manage profiles in injectors, with routine treatment in formations up to 150 °F. Acrylic-based polymers are a notable class of robust materials that perform well under challenging conditions in the reservoir. These polymers are typically synthesized through radical chain polymerization of acrylate monomers and are employed in various applications, including architectural, textile, and aircraft components [30,31,32,33,34,35]. By combining a high molecular weight acrylamide with a neutralized sodium acrylate, a novel crosslinked polymer can be synthesized to improve the overall performance of water and CO_2_ permeability reduction due to its acidic nature that could allow it to resist CO_2_.

Many studies have investigated the permeability reduction of both water and CO_2_ using crosslinked polymers, most of which focused on the utilization of the crosslinked polymer as water shutoff methods or CO_2_ shutoff methods separately. Cherepanova [35] developed a crosslinked polymer system for low temperature (12 °C) and high salinity (400 g/L) formation in Siberia. The crosslinked polymer was composed of a high-molecular-weight HPAM crosslinked with chromium salt. The study did not investigate the CO_2_ permeability reduction. Wang [36] tested a particle gel with a costly polymer for CO_2_ shutoff applications. They did not investigate the ability of the polymer to divert the flow of water. Additionally, their study only focused on fractures. Zhu [37] studied the utilization of a ter polymer with a PEI crosslinker for water conformance control in high-temperature formations. The ter polymer managed to divert the flow of water; however, the crosslinked polymer system was extremely costly. Also, the research did not investigate the CO_2_ permeability-reduction potential. Hatzignatiou [38] performed a screening of different crosslinked polymer systems composed primarily of sodium silicate. They investigated several properties of these polymers including gelant’s viscosity, pH, filterability, and injectivity; the gelation time and kinetics of the gelation process; strength of the formed gel against applied external forces; gel stability; gel shrinkage; and post-gelation-time behavior. They did not study the permeability-reduction potential of the crosslinked polymer for water or CO_2_. Choi [39] showed that HPAM-crosslinked polymers behaved differently under different pH conditions. This was an excellent indication that HPAM can be used under slightly acidic conditions, which allows for its utilization for CO_2_ permeability reduction. Deolarte [40] reported a crosslinked polymer system that was used successfully in different field applications for water shutoff, including water coning/cresting, high-permeability streaks, gravel-pack isolation, fracture shutoff, and casing-leak repair. The crosslinked system relied on a polymer and a PEI crosslinked. They did not study the potential of this crosslinked system to reduce the permeability of CO_2_.

Although many researchers have focused on the development of crosslinked polymers for water shutoffs, and some have investigated crosslinked polymers for CO_2_ shutoffs, very limited research has been reported for successful CO_2_ shutoff systems [41,42,43,44,45,46,47,48,49]. Additionally, very limited research has investigated the ability of a crosslinked polymer system to function under both CO_2_ and water conditions, which is vital for EOR applications that involve water alternating gas injection (WAG) and for CO_2_ storage applications in depleted oil reservoirs, which always have water in them. This research investigates the ability of particle crosslinked polymers to reduce the permeability of CO_2_ and brine for conformance control applications for EOR and CO_2_ sequestration. This research synthesizes two different crosslinker polymers using different monomers and tests their performance separately for comparison. The impact of different factors, including brine salinity, permeability of the formation, and the presence of crude oil, on the permeability-reduction performance of the particle crosslinked polymers is investigated.

## 2. Experimental Description

The polymer synthesis procedure, experimental material, setup, and procedure followed in conducting all the experiments are explained. The experimental design was made in order to develop and test the synthesized crosslinked polymers in a manner that would allow for the assessment of their ability to reduce water and CO_2_ permeability. This will help determine the overall objectives of the research, which is to improve the application of crosslinked polymers in waterflooding operations and CO_2_ EOR and storage applications.

### 2.1. Polymer Synthesis

**Figure 1:**
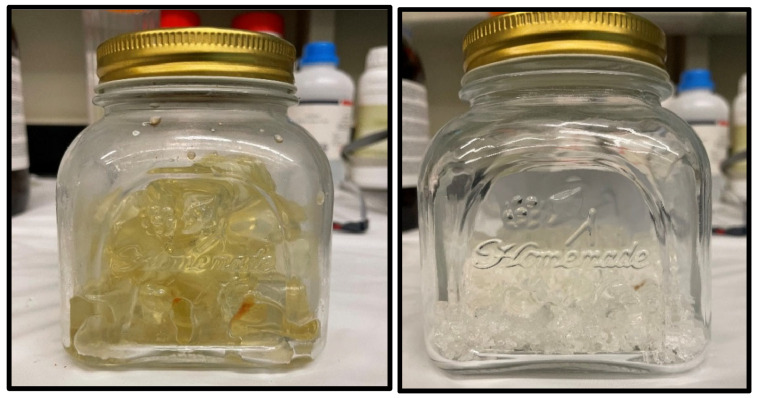
Synthesized AA/HPAM-crosslinked polymer (yellow) and HPAM-crosslinked polymer (transparent).

The two polymers were synthesized and crosslinked using the same process, with the only difference being the mechanics of crosslinking. All chemicals were provided from Sigma Aldrich with a purity of 99.9%. The following is the procedure followed to synthesize and crosslink the polymers shown in Figure 1:

- The monomer was mixed with distilled water using different ratios depending on the formulation. For the acrylic acid (AA), a neutralization reaction was conducted using 5 wt% sodium hydroxide (NaOH) to neutralize the solution. AA was provided as a clear liquid, while the NaOH was provided as white pellets.
- When the monomers were completely dissolved in the water, the crosslinker with a concentration of 0.01 wt% was added to the solution and thoroughly mixed. The crosslinker used was *N*,*N*’ Methylenebis(acrylamide), provided as a white powder for all mixtures.
- After adding the crosslinker, the solution was purged using nitrogen to remove all oxygen in the mixture. This is performed since oxygen has a significant impact on the integrity of the polymer. Purging was performed for two continuous hours with constant mixing using a magnetic stirrer.
- Finally, the initiator is added to begin the polymerization and the crosslinking process. The initiator used in all formulations was ammonium persulfate (APS), provided as white crystals.
- The solution is then left overnight in a water bath with a constant temperature of 35 °C. After that, the crosslinked polymer is cut into small pieces and placed in a desiccator to dehydrate. Following this, the particles are ground to uniform particles using a ball mill. They are sieved through a mesh screen to ensure uniform particle size distribution.

### 2.2. Experimental Material

The materials used to conduct all the experiments in this research are as follows:

- Acrylamide Monomer: the acrylamide monomer was provided as a white powder with high solubility in water at room temperature.
- Acrylic Acid: the acrylic acid was provided as a light-yellow liquid with a pungent odor.
- Sodium Hydroxide: sodium hydroxide was provided as white granular pellets.
- Distilled Water: this was used as the base for all experiments and was manufactured in the lab through a series of filtration processes.
- Ammonium per Sulfate: APS was provided as a white, flaky powder with 99.99% purity.
- Methylenebis(acrylamide): MBA was provided as a white powder with 99.99% purity.
- Sodium Chloride: NaCl was provided as transparent foggy granular solid pellets.
- Crude Oil: The oil used in this research was obtained as dead oil from an oil field in the western desert of Egypt. The composition of the crude oil was determined using chromatography and mass spectrometry and is presented in Table 1.
- Sand Particles: The sand particles were obtained from sandstone rock samples that were pulverized and sieved to obtain uniform distribution. The size was 50 mesh.

### 2.3. Experimental Setup

**Figure 2:**
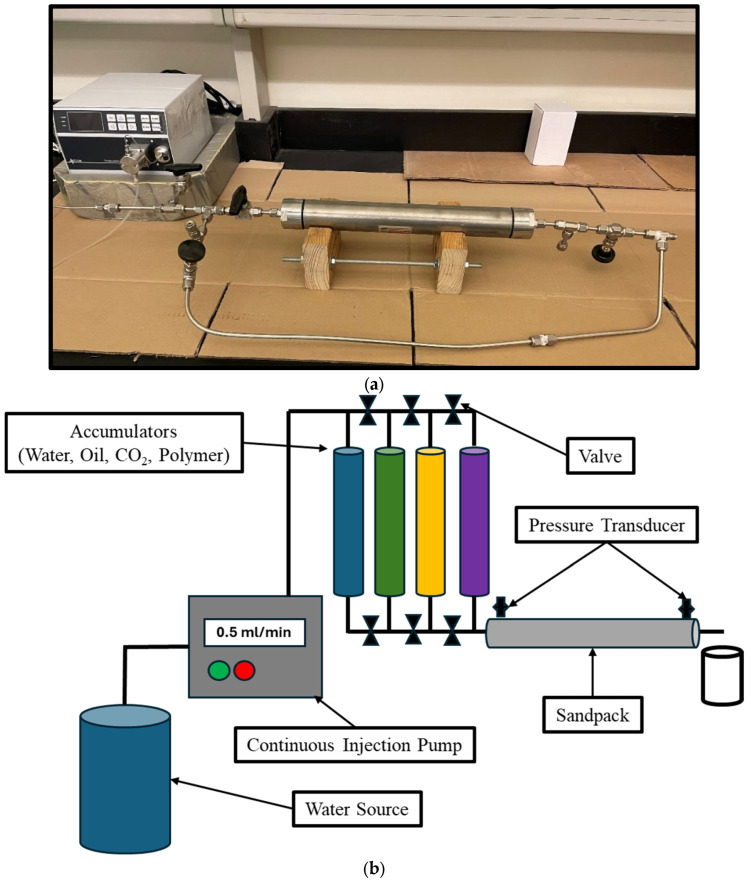
(**a**) Sandpack experimental setup. (**b**) Sandpack experimental setup.

The setup used to conduct the experiments is shown in Figure 2a, and an illustration of the setup is shown in Figure 2b. The setup is composed of a sandpack with a total length of 12.5 inches. The sandpack is equipped with a pressure transducer at the inlet and outlet to measure the pressure differential across the entire section. A high-pressure pump is connected to the accumulator to allow for the injection of the crosslinked polymer, brine, CO_2_, and crude oil. The pressure transducers are connected to a computer for pressure value recording and analysis.

### 2.4. Experimental Procedure

After polymer synthesis and preparation, the procedure followed to prepare the sandpack and run all the experiments is as follows:

- The sandpack was filled with sand of uniform size. It was packed tightly to ensure no air pockets existed. The sandpack was equipped with a mesh screen on both caps to prevent sand production during the experiments. The mesh screens were designed to avoid creating significant backpressure during injection. The sandpack was then vacuumed and sealed in preparation for the experiment.
- The sandpack permeability was measured by injecting water at a constant flow rate until the pressure stabilized. The water used was a brine solution with the same salinity as that of the chase water injected after the polymer.
- The polymer was prepared by swelling it in the design brine solution based on the experiment for 6 h to ensure full swelling. Following this, it was placed in an accumulator with excess water for ease of injection and to minimize shearing, syneresis, and dehydration.
- The experiment began by initially injecting the crosslinked polymer particles into the sandpack until the particles were observed at the outlet. Following this, the injection continued for another hour to ensure complete packing.
- The cycles of injection followed the polymer injection directly. The cycles included water followed by CO_2_, each injected at a rate of 0.02, 0.04, 0.05, and 0.07 bbl/day. For each rate, the injection continued until stabilized pressure was reached.
- The cycles continued until the permeability reduction varied slightly from one cycle to the other, or the reduction became extremely low to the point where the crosslinked polymer had no effect.

## 3. Results and Analysis

The experimental results for the impact of brine salinity, permeability variation, and crude oil interaction with the polymer on the ability of the crosslinker polymer to reduce the permeability of CO_2_ and formation brine are presented and explained. The permeability of the sandpack was varied by artificially altering the packing of the grains within the sandpack. Based on this, two different permeability values were used, including a 3 Darcy sandpack with good packing and an 18 Darcy sandpack with loose packing. For all experiments, the sandpack permeability used was 3 Darcy. The only experiment that used the 18 Darcy sandpack was the experiment that tested the increase in porous media permeability.

### 3.1. CO_2_ Permeability Reduction

CO_2_ conformance control is extremely important not only for CO_2_ EOR applications but also for CO_2_ sequestration wells. The impact of brine salinity, permeability variation, and the presence of crude oil on the ability of the crosslinked polymer to reduce the permeability of CO_2_ is explained. The decrease in permeability reduction with different cycles was a direct indication of the crosslinked polymer degradation through different mechanisms, including shearing and dehydration.

#### 3.1.1. Impact of Brine Salinity

The impact of brine salinity on the CO_2_ permeability-reduction potential of the crosslinked polymers was studied using 1% and 10% by weight of NaCl. Increasing salinity results in a decrease in the ability of the crosslinked polymer to absorb water. Based on this, the crosslinked polymer will swell less, and the particles will have a smaller volume. This will result in the polymer particles having a higher strength compared to the particles placed in a lower-concentration brine. This can improve their ability to withstand higher-pressure differentials. However, it also reduces their elasticity significantly, which results in higher injection pressure.

**Figure 3:**
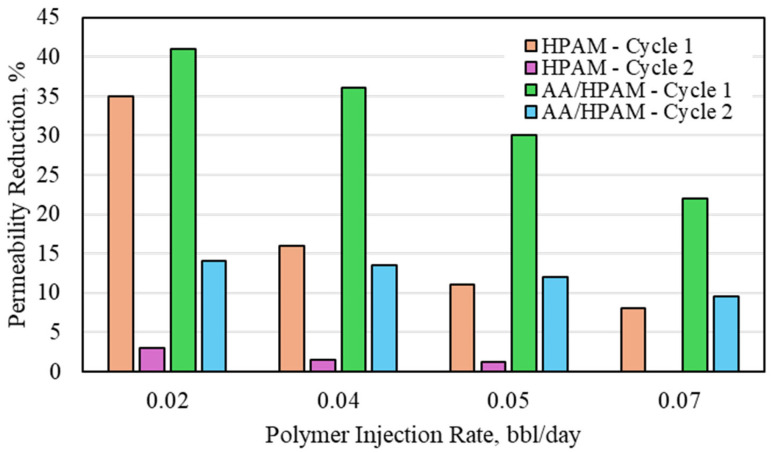
CO_2_ permeability reduction for HPAM- and AA/HPAM-crosslinked polymer after one and two injection cycles at different injection rates using 3 Darcy permeability sandpack, 1 wt% polymer, and 1 wt% NaCl.

The CO_2_ permeability reduction for HPAM- and AA/HPAM-crosslinked polymers after one and two injection cycles at different injection rates using a 3 Darcy permeability sandpack, 1 wt% polymer, and 1 wt% NaCl is shown in Figure 3. For both polymers, as the injection rate of the CO_2_ increased, the permeability-reduction percent decreased. This was due to the increase in pressure of the CO_2_ as the injection rate increased, which, in turn, caused polymer shearing and syneresis. This was observed from the crosslinked polymer sample collected from the outlet during CO_2_ injection. The HPAM polymer sample had a relatively low permeability reduction, and it reduced even more with the second injection cycle. It reached a value of less than 1%, which indicates almost total degradation during the second cycle of the injection rate of 0.07 bbl/day. The AA/HPAM-crosslinked copolymer outperformed the AA polymer, with a maximum permeability reduction of 42.5%. Its performance, however, was still reduced significantly during the second cycle, with a minimum permeability of 9.8%. Overall, the AA/HPAM had a much higher level of performance. This is primarily attributed to the AA being acidic in nature and, therefore, much more resistant to CO_2_ compared to the HPAM.

**Figure 4:**
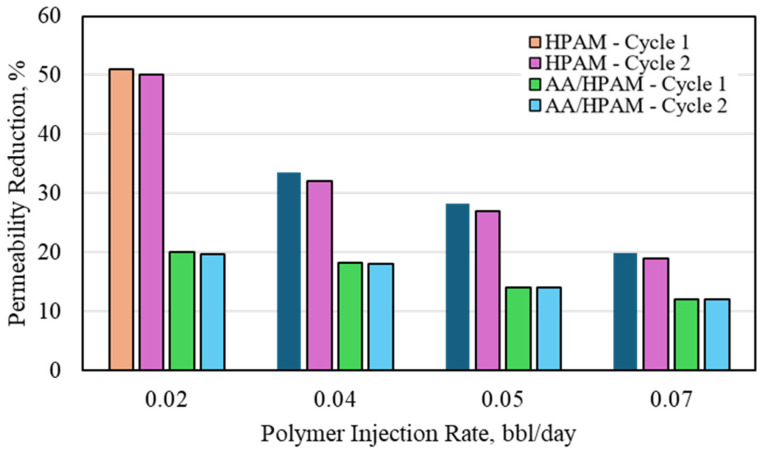
CO_2_ permeability reduction for HPAM- and AA/HPAM-crosslinked polymers after one and two injection cycles at different injection rates using 3 Darcy permeability sandpack, 1 wt% Polymer, and 10 wt% NaCl.

The CO_2_ permeability reduction for HPAM- and AA/HPAM-crosslinked polymers after one and two injection cycles at different injection rates using a 3 Darcy permeability sandpack, 1 wt% polymer, and 10 wt% NaCl is shown in Figure 4. Contrary to the 1% NaCl experiments, the HPAM outperformed the AA/HPAM-crosslinked polymer when 10% NaCl brine was used. The HPAM showed more than 50% permeability reduction and decreased very slightly during the second cycle. Increasing the injection rate reduced the permeability reduction; however, with the cycle progression, the polymer showed very little impact. The same trend was observed with the HPAM/AA-crosslinked polymer; however, it exhibited a much lower overall permeability reduction. The reason behind this was observed during the crosslinked particle swelling ratio experiments for both polymers. Using the same initial dry particle size, when both polymers were swollen in a 1% NaCl solution, the swelling ratio of the AA/HPAM particle was slightly higher than that of the HPAM. When the same experiment was repeated for the 10% NaCl experiment, the AA/HPAM particle swelled much more, which indicated that it was impacted less by salinity compared to the HPAM. This resulted in the HPAM particles becoming stronger due to lower swelling compared to the AA/HPAM particles, which, in turn, resulted in a higher permeability reduction. Both polymers were not impacted significantly by the cycle progression due to the high strength that they exhibited compared to the 1% NaCl brine.

#### 3.1.2. Impact of Permeability Variation

Crosslinked polymer propagation through porous media is impacted by rock particles in different manners. When the permeability of the rock is increased, the particles will move more freely through the pores, which will result in a reduction in the injection pressure gradients. This will reduce polymer shearing and dehydration significantly; however, it will also impact the permeability-reduction potential of the polymer greatly since it will become much more difficult for the polymer to plug the larger pores in a state of higher permeability. When the permeability of the porous media is lowered, the polymer permeability-reduction potential is improved significantly; however, polymer injection requires higher pressures. Also, the polymer will be more prone to dehydration and shearing. This can impact the ability of the crosslinked polymer to be used for CO_2_ permeability reduction in EOR applications and CO_2_ storage applications, which require extremely high integrity for the polymers [50].

**Figure 5:**
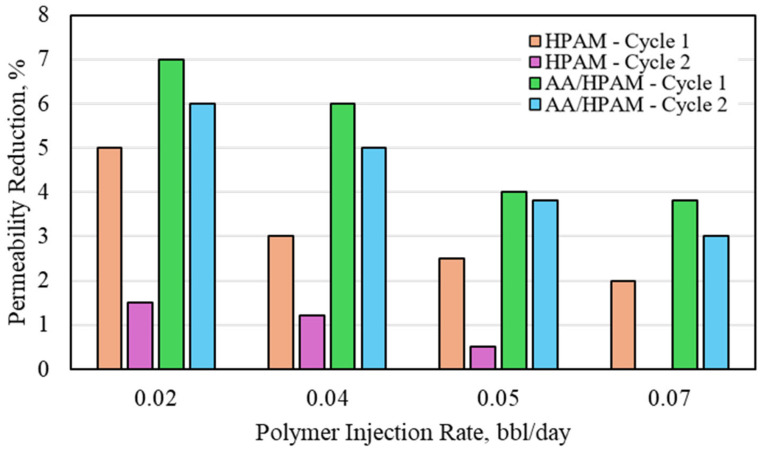
CO_2_ permeability deduction for HPAM- and AA/HPAM-crosslinked polymers after one and two injection cycles at different injection rates using 18 Darcy permeability sandpack, 1 wt% Polymer, and 1 wt% NaCl.

The CO_2_ permeability reduction for HPAM- and AA/HPAM-crosslinked polymers after one and two injection cycles at different injection rates using a 3 Darcy permeability sandpack, 1 wt% polymer, and 1 wt% NaCl is presented in Figure 3. Figure 5 shows the CO_2_ permeability reduction for HPAM- and AA/HPAM-crosslinked polymers under the same conditions but with a permeability of 18 Darcy. The permeability of the sandpack was altered by varying the sand particle size and sorting. When the permeability of the sandpack was increased, the CO_2_ permeability reduction of both polymers was reduced significantly, reaching a maximum of only 7%. As for the general behavior, it is similar to what was observed in the 3 Darcy experiment, with the AA/HPAM-crosslinked polymer outperforming the HPAM. Overall, however, both crosslinker polymers performed very poorly due to the significantly high permeability of the sandpack.

#### 3.1.3. Impact of Crude Oil–Polymer Interaction

**Figure 6:**
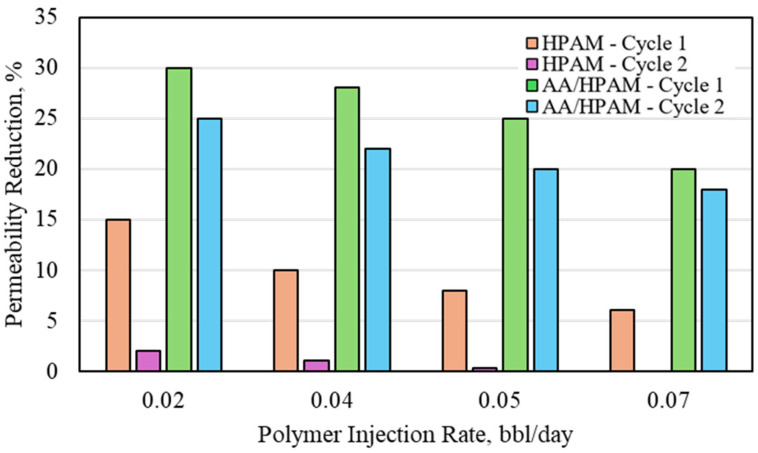
CO_2_ permeability reduction for HPAM- and AA/HPAM-crosslinked polymers after one and two injection cycles at different injection rates using 3 Darcy permeability sandpack, 1 wt% polymer, 1 wt% NaCl, and 33 cp Crude Oil.

Since the crosslinked polymers have the ability to absorb fluid, they have the potential to absorb some oil. This may impact the properties of the polymers. Crude oil was introduced in the sandpack by injecting the oil after measuring the sandpack permeability in the initial stage. Following the crude oil injection, chase water was injected until no more oil was produced. After this, the crosslinked polymer was injected, and then the CO_2_-water cycles began. The impact of crude oil on the CO_2_ permeability reduction for HPAM- and AA/HPAM-crosslinked polymers after one and two injection cycles at different injection rates using a 3 Darcy permeability sandpack, 1 wt% polymer, and 1 wt% NaCl is shown in Figure 6. Based on the results, the presence of crude oil had a significant impact on the CO_2_ permeability reduction of the HPAM-crosslinked polymer, where its permeability was almost halved compared to the same experiment with no crude oil. When the HPAM particles were collected and analyzed after the experiment, it was found that the samples absorbed a small percentage of the crude oil. The particles became extremely fragile and exhibited severe and significant hydrolysis. On the other hand, the AA/HPAM-crosslinked polymer was not strongly impacted by the presence of crude oil. This could be due to the presence of AA; since both the crude oil and the AA are acidic in nature, the crude oil did not impact the AA greatly. The CO_2_ permeability potential of AA/HPAM-crosslinked polymer was still slightly impacted due to the presence of HPAM in the crosslinked copolymer structure.

### 3.2. Formation Water Permeability Reduction

Crosslinked polymers have been used extensively as conformance control agents. This research focuses on the ability of both HPAM- and AA/HPAM-crosslinked polymers to reduce the permeability of formation water with 1% and 10% NaCl salinity when subjected to multiple CO_2_ and water injection cycles. This will test the durability of the polymer, especially during applications that involve both fluids, such as water alternating gas injection.

#### 3.2.1. Impact of Brine Salinity

**Figure 7:**
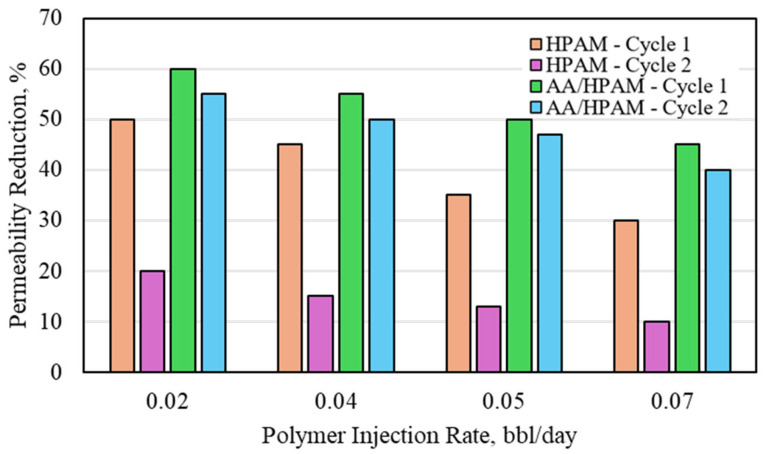
Water permeability reduction for HPAM- and AA/HPAM-crosslinked polymers after one and two injection cycles at different injection rates using 3 Darcy permeability sandpack, 1 wt% Polymer, and 1 wt% NaCl.

As mentioned previously and as observed in multiple pieces of research, brine will have an impact on the swelling capacity and the strength of crosslinked polymers [3,4,5,6,7,8,9,10,11,12,13,14,15,16,17,18]. The water permeability reduction for HPAM- and AA/HPAM-crosslinked polymers after one and two injection cycles at different injection rates using a 3 Darcy permeability sandpack, 1 wt% polymer, and 1 wt% NaCl is shown in Figure 7. The AA/HPAM polymer had a slightly higher permeability-reduction potential compared to the HPAM polymer. The main advantage of the AA/HPAM polymer over the HPAM polymer is during the second cycle of injection, where the AA/HPAM polymer has a much lower degradation compared to the HPAM polymer.

**Figure 8:**
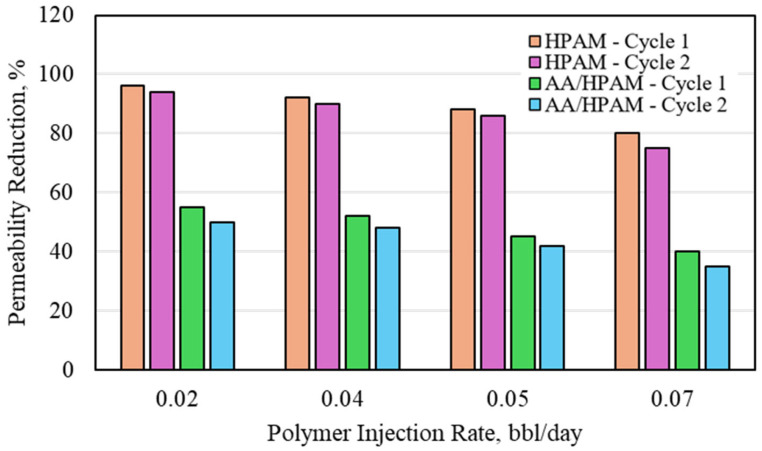
Water permeability reduction for HPAM- and AA/HPAM-crosslinked polymers after one and two injection cycles at different injection rates using 3 Darcy permeability sandpack, 1 wt% Polymer, and 10 wt% NaCl.

The water-permeability reduction for HPAM- and AA/HPAM-crosslinked polymers after one and two injection cycles at different injection rates using a 3 Darcy permeability sandpack, 1 wt% polymer, and 10 wt% NaCl is shown in Figure 8. When the salinity was increased, the same observation was seen as in the CO_2_ experiment. The HPAM polymer has a better performance due to its lower level of water absorption compared to the AA/HPAM polymer. The HPAM polymer reached its maximum permeability-reduction value, reaching 96% at the lower injection rate of 0.02 bbl/day for the first cycle and 94% for the second cycle. The AA/HPAM polymer exhibited a permeability reduction of more than 50% for the same cycle and rate, which is much higher than in the CO_2_ experiment. All in all, the permeability-reduction potential for the formation water using both crosslinked polymers was much higher than the CO_2_ permeability-reduction potential. This is mainly attributed to the flow behavior of the CO_2_ and its acidic properties compared to the formation water used to conduct the experiments.

#### 3.2.2. Impact of Permeability Variation

**Figure 9:**
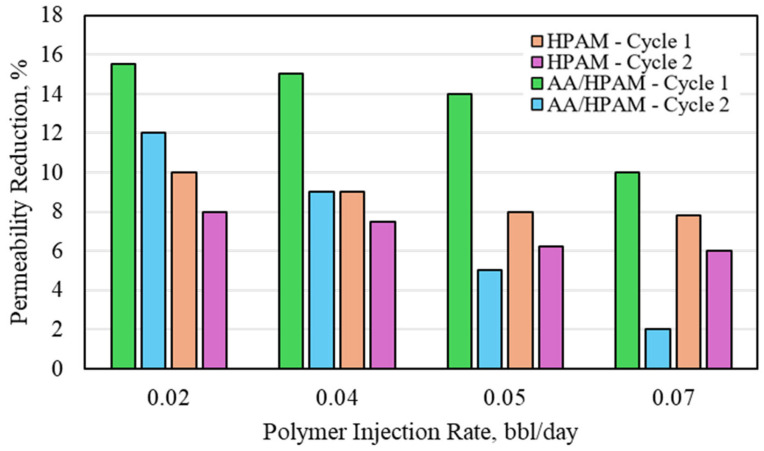
Water permeability reduction for HPAM- and AA/HPAM-crosslinked polymers after one and two injection cycles at different injection rates using 18 Darcy permeability sandpack, 1 wt% polymer, and 1 wt% NaCl.

Similar to the CO_2_ permeability-reduction experiments, the permeability of the sandpack was increased to investigate its impact on the formation water permeability reduction for both crosslinked polymers. The water permeability reduction for HPAM- and AA/HPAM-crosslinked polymers after one and two injection cycles at different injection rates using an 18 Darcy permeability sandpack, 1 wt% polymer, and 1 wt% NaCl is shown in Figure 9. When the permeability was increased, the permeability-reduction potential for both crosslinked polymers was reduced significantly, reaching less than 20% for the highest value, compared to more than 90% for the 3 Darcy experiment. The decrease in performance is similar to that observed in studies that utilized crosslinked polymer for fractures and high permeability features for conformance control [22,23,24,25,26,27,28,29,30,31,32,33,34,35,36]. The performance of both crosslinked polymers was, however, much higher compared to the CO_2_ experiments. The AA/HPAM polymer had a higher permeability reduction compared to the HPAM polymer due to its higher water-absorption capacity and higher swelling ratio.

#### 3.2.3. Impact of Crude Oil–Polymer Interaction

**Figure 10:**
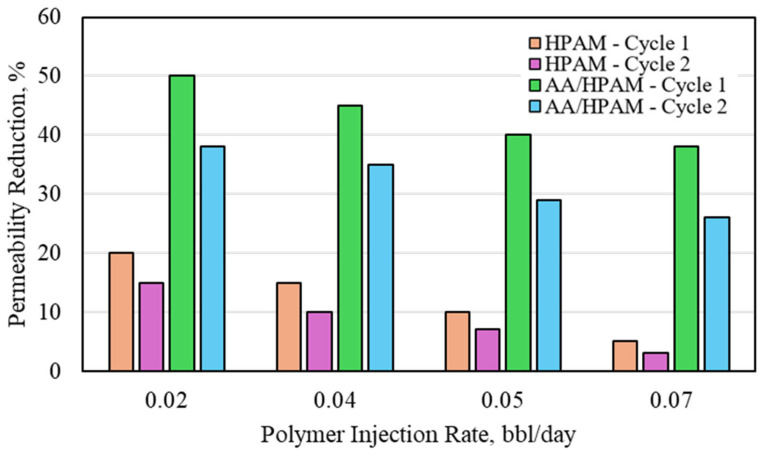
Water permeability reduction for HPAM- and AA/HPAM-crosslinked polymers after one and two injection cycles at different injection rates using 3 Darcy permeability sandpack, 1 wt% polymer, 1 wt% NaCl, and 33 cp crude oil.

The impact of crude oil on the water permeability reduction for HPAM- and AA/HPAM-crosslinked polymers after one and two injection cycles at different injection rates using a 3 Darcy permeability sandpack, 1 wt% polymer, and 1 wt% NaCl is shown in Figure 10. The same impact occurred as in the CO_2_ experiments, with the AA/HPAM polymer outperforming the HPAM polymer. The performance of both crosslinked polymers was, however, higher compared to the CO_2_ experiments. Due to its acidic nature, the crude oil had some impact on the polymer network, although the HPAM/AA polymer was impacted very little due to the acidic properties of the AA; this was reported by several studies that utilized acidic-based polymers or copolymers [45,46,47,48,49]. Based on this, it can be concluded that, for all experiments, the ability of both the crosslinked polymers to reduce the permeability of water was much better than their CO_2_ permeability-reduction potential.

## 4. Limitations and Future Work

This research synthesized and tested some properties of two crosslinked polymers composed of HPAM and HPAM/AA for water and CO_2_ permeability reduction. Some of the main limitations of this research that require further study and will be part of future work for this research include the following tests. A complete analysis of the crosslinked polymers’ properties before and after injection should be investigated. This is currently being conducted as part of the future work for this project. The analysis will include swelling and deswelling ratios for several cycles, gel strength, crosslinked polymer pH before and after subjection to formation water and CO_2_, viscosity, filterability to determine the ability to extrude through small pores without excessive syneresis, injectivity in different pore sizes, gelation time using different polymer and crosslinker concentrations, kinetics of gelation process, gel stability for long durations, gel shrinkage, post-injection gel behavior, crosslinked polymer syneresis and dehydration, polymer shearing, and polymer network alteration through SEM.

## 5. Conclusions

This research investigates the ability of two crosslinked polymers, including HPAM and AA/HPAM polymers, to reduce the permeability of both CO_2_ and formation water under different conditions. This could assist in improving oil recovery from waterflooding operations and CO_2_ EOR, including CO_2_ injection and water alternating CO_2_ injection. It could also be a means of improving CO_2_ storage volume in depleted oil and gas reservoirs. The main conclusions obtained from this research are as follows:

- Field applications of crosslinked polymers using HPAM and AA can be effectively applied in waterflooding and CO_2_ EOR and storage applications; however, a thorough analysis of the reservoir rock, fluid, and thermodynamic properties must be conducted.
- For HPAM- and AA-crosslinked polymers, the permeability reduction for water is extremely effective, whereas the permeability reduction for CO_2_ requires further improvement to be able to apply this technology under a broader range of conditions.
- Increasing the brine concentration had a positive impact on the HPAM-crosslinked polymer, with an increase in its strength and permeability-reduction potential, whereas the AA/HPAM degraded slightly. This is primarily due to the impact of monovalent and divalent cations on the polymers’ ability to absorb water.
- Extremely high permeability values for the porous media are very challenging for crosslinked particle polymers to plug, and, therefore, the permeability-reduction potential for both crosslinked polymers was very low. This could be overcome by increasing the polymer concentration; however, this would be extremely costly.

## Figures and Tables

**Table 1:** Crude oil composition.

Component	Weight Percent, %
C1–C5	9.37
C6–C10	14.74
C11–C15	18.89
C16–C20	26.31
C21–C30	11.63
C30+	19.06

## Data Availability

The raw data supporting the conclusions of this article will be made available by the authors upon request.

## References

[1] Bariki R., Joseph R.G., El-Kadri O.M., Al-Sayah M.H. (2024). The Development of Metal-Free Porous Organic Polymers for Sustainable Carbon Dioxide Photoreduction. Nanomaterials.

[2] Wu M., Liu W., Ma Z., Qin T., Chen Z., Zhang Y., Cao N., Xie X., Chi S., Xu J. (2024). Global Trends in the Research and Development of Petrochemical Waste Gas from 1981 to 2022. Sustainability.

[3] Pagáč M., Opletal V., Shchipanov A., Nermoen A., Berenblyum R., Fjelde I., Rez J. (2024). Integrated Approach to Reservoir Simulations for Evaluating Pilot CO_2_ Injection in a Depleted Naturally Fractured Oil Field On-Shore Europe. Energies.

[4] Worden R.H. (2024). Carbon Dioxide Capture and Storage (CCS) in Saline Aquifers versus Depleted Gas Fields. Geosciences.

[5] Shakiba M., Ghomi E.R., Khosravi F., Jouybar S., Bigham A., Zare M., Abdouss M., Moaref R., Ramakrishna S. (2021). A Material Introduction and Overview for Biomedical Applications. Polym. Adv. Technol..

[6] Flory P.J. (1941). Molecular Size Distribution in Three Dimensional Polymers. I. Gelation1. J. Am. Chem. Soc..

[7] Das D.B. (2024). CO_2_ Capture and Sequestration. Clean Technol..

[8] Gupta A., Paul A.R., Saha S.C. (2023). Decarbonizing the Atmosphere Using Carbon Capture, Utilization, and Sequestration: Challenges, Opportunities, and Policy Implications in India. Atmosphere.

[9] Zhou W., Pan L., Mao X. (2023). Optimization and Comparative Analysis of Different CCUS Systems in China: The Case of Shanxi Province. Sustainability.

[10] Fakher S., Khlaifat A., Hassanien A. (2024). Carbon Dioxide Capture under Low-Pressure Low-Temperature Conditions Using Shaped Recycled Fly Ash Particles. Gases.

[11] Khan M.K.A., Khan J.A., Ullah H., Al-Kayiem H.H., Irawan S., Irfan M., Glowacz A., Liu H., Glowacz W., Rahman S. (2021). De-Emulsification and Gravity Separation of Micro-Emulsion Produced with Enhanced Oil Recovery Chemicals Flooding. Energies.

[12] Haydar R., Fakher S. Harsh Environmental Effects on Low Density Fly Ash Proppants. Proceedings of the Mediterranean Offshore Conference.

[13] Salib A.M., Fakher S. Development and Optimization of Epoxy-Resin Based Cement Reinforced with Low-Cost Fly Ash for High Durability Environmentally Friendly Cement. Proceedings of the Mediterranean Offshore Conference.

[14] Elsayed A., Fakher S. Investigating Asphaltene Damage Reduction in Wellbores, and Pipelines Using Alkaline and Surfactant Chemical Agents. Proceedings of the Mediterranean Offshore Conference.

[15] Helmy Y., Fakher S. (2024). Evaluating the Performance of Class F Fly Ash Compared to Class G Cement for Hydrocarbon Wells Cementing: An Experimental Investigation. Materials.

[16] Jin F.-L., Li X., Park S.-J. (2015). Synthesis and Application of Epoxy Resins: A Review. J. Ind. Eng. Chem..

[17] Chan J.W., Shin J., Hoyle C.E., Bowman C.N., Lowe A. (2010). Synthesis, Thiol—Yne “Click” Photopolymerization, and Physical Properties of Networks Derived from Novel Multifunctional Macromolecules. Macromolecules.

[18] Khamees T.K., Flori R.E., Fakher S.M. Numerical Modeling of Water-Soluble Sodium Silicate Gel System for Fluid Diversion and Flow-Zone Isolation in Highly Heterogeneous Reservoirs. Proceedings of the SPE Trinidad and Tobago Section Energy Resources Conference.

[19] Fakher S., Abdelaal H., Elgahawy Y., Imqam A. (2019). A characterization of different alkali chemical agents for alkaline flooding enhanced oil recovery operations: An experimental investigation. SN Appl. Sci..

[20] Numin M.S., Jumbri K., Ramli A., Borhan N. (2020). Microemulsion Rheological Analysis of Alkaline, Surfactant, and Polymer in Oil-Water Interface. Processes.

[21] Khlaifat A., Fakher S., Ibrahim A.D., Elsese M., Nour A. (2023). High-salinity produced water treatment and desalination. LHB.

[22] Hoskin E.M., Mahyildin S., Crookes M. Quantitative Assessment of End-of-Life Wells and Fields for Carbon Captureand Storage (CCS) Suitability. Proceedings of the SPE/IADC Asia Pacific Drilling Technology Conference and Exhibition.

[23] Fakher S., Khlaifat A. (2022). Gas Slippage in Tight Formation. Topic on Oil and Gas.

[24] Khlaifat A.L. (2021). Displacement Efficiency in Tight Sandstone Based on Fractional Flow Curve Using Relative Permeability Data. J. Geotechnol. Energy.

[25] Khlaifat A.L., Fakher S., Harrison G.H. (2024). Evaluating Factors Impacting Polymer Flooding in Hydrocarbon Reservoirs: Laboratory and Field-Scale Applications. Polymers.

[26] Dai C., Ge J., Zhang G., Zhao F. (2001). Study on factors affecting the freezing of zirconium jelly. Oilfield Chem..

[27] Cui J. (2017). Preparation and performance evaluation of zirconium jelly dispersion modifier. Oilfield Chem..

[28] Li B., Yao E. High-temperature resistant, low-concentration polyacrylamidee gel system. American Rock Mechanics Association. Proceedings of the 56th US Rock Mechanics/Geomechanics Symposium.

[29] Crespo F., Reddy B.R., Eoff L. Christopher Lewis, and Natalie Pascarella. Development of a Polymer Gel System for Improved Sweep Efficiency and Injection Profile Modification of IOR/EOR Treatments. Proceedings of the International Petroleum Technology Conference.

[30] DiGiacomo P.M., Schramm C.M. Mechanism of Polyacrylamide Gel Syneresis Determined by C-13 NMR. Proceedings of the International Symposium on Oilfield and Geothermal Chemistry.

[31] Odian G. (2004). Principles of Polymerization.

[32] Johnson L.M., Shepherd S.D., Rothrock G.D., Cairns A.J., Al-Muntasheri G.A. Core-Shell Systems for Delayed Delivery of Concentrated Mineral Acid. Proceedings of the SPE International Symposium on Oilfield Chemistry.

[33] Seright R.S. (2017). How much polymer should be injected during a polymer flood? Review of previous and current practices. SPE J..

[34] Standnes D.C., Skjevrak I. (2014). Literature review of implemented polymer field projects. J. Pet. Sci. Eng..

[35] Cherepanova N.A., Kochetov A.V., Tagirov K.D., Krever A.S., Ivanov E.N., Kopylov A.V. (2023). Justifying the applicability of conformance control technologies in terrigenous reservoirs of Eastern Siberia. OIJ.

[36] Wang Z., Bai B., Long Y., Wang L. (2019). An Investigation of CO_2_-Responsive Preformed Particle Gel for Conformance Control of CO_2_ Flooding in Reservoirs with Fractures or Fracture-Like Channels. SPE J..

[37] Zhu D., Hou J., Chen Y., Wei Q., Zhao S., Bai B. (2019). Evaluation of Terpolymer-Gel Systems Crosslinked by Polyethylenimine for Conformance Improvement in High-Temperature Reservoirs. SPE J..

[38] Hatzignatiou D.G., Askarinezhad R., Giske N.H., Stavland A. (2016). Laboratory Testing of Environmentally Friendly Sodium Silicate Systems for Water Management Through Conformance Control. SPE Prod. Oper..

[39] Choi S.K., Sharma M.M., Bryant S.L., Huh C. (2010). pH-Sensitive Polymers for Novel Conformance-Control and Polymer-Flood Applications. SPE Res. Eval. Eng..

[40] Deolarte C., Vasquez J.E., Soriano E., Santillan A. (2009). Successful Combination of an Organically Crosslinked Polymer System and a Rigid-Setting Material for Conformance Control in Mexico. SPE Prod. Oper..

[41] Wei B., Chen S., Tian Q., Lu J., Xu X. (2020). In-Situ Generation and Propagation of Well-Defined Nanocellulose Strengthened Gaseous and Supercritical Carbon Dioxide Foams in Tight Formation Fractures for Conformance Control. SPE Res. Eval. Eng..

[42] Salman M., Kostarelos K., Sharma P., Lee J.H. (2020). Application of Miscible Ethane Foam for Gas EOR Conformance in Low-Permeability Heterogeneous Harsh Environments. SPE J..

[43] Torrealba V.A., Hoteit H. (2019). Conformance Improvement in Oil Reservoirs by Use of Microemulsions. SPE Res. Eval. Eng..

[44] Zhu D., Hou J., Wei Q., Chen Y. (2019). Development of a High-Temperature-Resistant Polymer-Gel System for Conformance Control in Jidong Oil Field. SPE Res. Eval. Eng..

[45] Izgec O., Shook G.M. (2012). Design Considerations of Waterflood Conformance Control with Temperature-Triggered, Low-Viscosity Submicron Polymer. SPE Res. Eval. Eng..

[46] Eoff L.S., Dalrymple D., Everett D.M., Vasquez J.E. (2007). Worldwide Field Applications of a Polymeric Gel System for Conformance Applications. SPE Prod. Oper..

[47] Taabbodi L., Asghari K. (2006). Application of In-Depth Gel Placement for Water and Carbon Dioxide Conformance Control in Carbonate Porous Media. J. Can. Pet. Technol..

[48] Reddy B.R., Eoff L.S., Dalrymple E.D., Brown D.L. (2005). Natural Polymer-Based Compositions Designed for Use in Conformance Gel Systems. SPE J..

[49] Bani E., Tremblay B., Polikar M., Wiwchar B., Huang H. (2004). Rheology of Sandy Polyacrylamide Gels: Application to Conformance in Cold Production. J. Can. Pet. Technol..

[50] Kazemzadeh E., Fuinhas J.A., Salehnia N., Koengkan M., Silva N. (2023). Exploring necessary and sufficient conditions for carbon emission intensity: A comparative analysis. Environ. Sci. Pollut. Res..

